# Emerging resistance among bacterial pathogens in the intensive care unit – a European and North American Surveillance study (2000–2002)

**DOI:** 10.1186/1476-0711-3-14

**Published:** 2004-07-29

**Authors:** Mark E Jones, Deborah C Draghi, Clyde Thornsberry, James A Karlowsky, Daniel F Sahm, Richard P Wenzel

**Affiliations:** 1 Focus Technologies, Herndon, Virginia, USA 20171; 2 Virginia Commonwealth University, Richmond, Virginia, USA

**Keywords:** Intensive-care unit, antibiotic susceptibility

## Abstract

**Background:**

Globally ICUs are encountering emergence and spread of antibiotic-resistant pathogens and for some pathogens there are few therapeutic options available.

**Methods:**

Antibiotic in vitro susceptibility data of predominant ICU pathogens during 2000–2 were analyzed using data from The Surveillance Network (TSN) Databases in Europe (France, Germany and Italy), Canada, and the United States (US).

**Results:**

Oxacillin resistance rates among *Staphylococcus aureus* isolates ranged from 19.7% to 59.4%. Penicillin resistance rates among *Streptococcus pneumoniae* varied from 2.0% in Germany to as high as 20.2% in the US; however, ceftriaxone resistance rates were comparably lower, ranging from 0% in Germany to 3.4% in Italy. Vancomycin resistance rates among *Enterococcus faecalis* were ≤ 4.5%; however, among *Enterococcus faecium* vancomycin resistance rates were more frequent ranging from 0.8% in France to 76.3% in the United States. Putative rates of extended-spectrum β-lactamase (ESBL) production among *Enterobacteriaceae* were low, <6% among *Escherichia coli* in the five countries studied. Ceftriaxone resistance rates were generally lower than or similar to piperacillin-tazobactam for most of the *Enterobacteriaceae* species examined. Fluoroquinolone resistance rates were generally higher for *E. coli* (6.5% – 13.9%), *Proteus mirabilis* (0–34.7%), and *Morganella morganii* (1.6–20.7%) than other *Enterobacteriaceae* spp (1.5–21.3%). *P. aeruginosa* demonstrated marked variation in β-lactam resistance rates among countries. Imipenem was the most active compound tested against *Acinetobacter* spp., based on resistance rates.

**Conclusion:**

There was a wide distribution in resistance patterns among the five countries. Compared with other countries, Italy showed the highest resistance rates to all the organisms with the exception of *Enterococcus* spp., which were highest in the US. This data highlights the differences in resistance encountered in intensive care units in Europe and North America and the need to determine current local resistance patterns by which to guide empiric antimicrobial therapy for intensive care infections.

## Background

Antimicrobial resistance has emerged as an important factor in predicting outcomes and overall resource use after infections in intensive care units (ICU) [1]. Globally ICUs are encountering emergence and spread of antibiotic-resistant pathogens. For some pathogens there are few therapeutic options available, e.g., vancomycin-resistant *Enterococcus faecium*. Awareness of these problems has been underscored with data from a number of surveillance studies aimed at improving the use of empiric therapy. In the United States there have been several national programs, which have focused on both the etiology of infections and resistance patterns of nosocomial or ICU infections including the National Nosocomial Infections Surveillance (NNIS) [2] and more recently an ICU-specific study examining the epidemiology of antimicrobial resistance, Project ICARE [3,4]. Stephen et al. collected strains from 28 ICUs from across the United States as part of the SENTRY Antimicrobial Surveillance Program in 2001 [5].

European data on the antimicrobial resistance of ICU pathogens has also been collected in several recent surveillance studies. A large prevalence survey of nosocomial infections in ICUs in 17 countries was published in 1995 [6], and more recently a number of nation-specific surveys were reported [7-9]. Several key points emerge: first, antimicrobial resistance among ICU pathogens is generally increasing, but variations do exist among different countries, probably due to individual antimicrobial use patterns; second, when new medical practices and alternative antimicrobials are introduced changes in the dominant microbial etiologies may emerge prompting novel empiric selections; and third, the standards of hygiene and infection control also vary across countries. Finally, appropriate therapy of ICU infections directed by local resistance data can have significant consequences for both patient and the healthcare system. It is against this background that local resistance surveillance programs are of most value in developing appropriate therapeutic guidelines for specific infections and patient types. For example, the recent modification to the American Thoracic Society guidelines for the treatment of hospital-acquired pneumonia [10] considered contemporary resistance data. Local surveillance data can be applied to other infections to assist in local formulary policy such as those governing treatment of nosocomial urinary tract infections [11].

This study using TSN program reports the antimicrobial resistance profiles of bacterial isolates from ICU patients in five countries during the period 2000–2002. The relevance of these recent nation-specific data will be discussed on a country-by-country basis, as part of improving and updating empiric therapeutic approaches to specific pathogens causing infections in the ICU setting according to each country. These surveillance programs help to maintain current knowledge of susceptibilities and relevant treatment options.

## Methods

### TSN Database – United States and Europe

TSN is a queriable, real-time database that electronically assimilates daily antimicrobial susceptibility testing and patient demographic data from a network of geographically dispersed laboratories in the United States (283 hospital sites), France (63 hospital sites), Germany (169 hospital sites), Italy (48 hospital sites) and Canada (87 hospital sites) [12].

Laboratories included in TSN include those servicing university, community, and private hospitals with bed sizes ranging from 100 to >1000 beds. Routine diagnostic susceptibility testing results are collected daily from each participating laboratory. The methods used by these laboratories include VITEK (bioMérieux, St. Louis, MO), MicroScan (Dade-Microscan, Sacramento, CA), Sceptor and Pasco MIC/ID (Becton Dickinson, Sparks, MD) and Etest (AB Biodisk, Solna, Sweden), as well as manual broth microdilution MIC, disk diffusion and agar dilution. TSN reflects current testing in participant laboratories and represents the data reported to physicians from the respective laboratories [13].

Although some European countries have alternate breakpoints, all data forwarded to TSN Databases are derived from hospitals that utilized NCCLS standards and definitions (United States, Canada, Italy, and Germany) [14] or the Comité de L'Antibiogramme de La Societé Français de Microbiologie (France) [15] thus standardizing datasets. Results were interpreted as susceptible, intermediate (if available), or resistant in TSN, based upon the NCCLS interpretative guidelines in place during 2001 [16]. In addition, a series of quality-control filters (i.e., critical rule sets) were used in TSN to screen susceptibility test results for patterns indicative of testing error and suspect results were removed from analysis for laboratory confirmation. In TSN, any result from the same patient with the same organism identification and the same susceptibility pattern received within five days was considered a repeat culture and was counted only once in the database.

### Bacterial species and antimicrobials tested

For this study, data from TSN results for each individual database from January 1, 2000 through to December 31, 2002 were included in the analysis to determine the proportion of species and their susceptibility to antimicrobial agents commonly tested in clinical laboratories throughout the participating regions. Only isolates derived from patients located in hospital ICUs were considered in the analysis. Gram-positive species included in the analysis were comprised of *S. aureus*, coagulase negative staphylococci, *Enterococcus faecalis*,*Enterococcus faecium*, *Streptococcus pyogenes*, *Streptococcus pneumoniae* and viridans group streptococci. Gram-negative species studied comprised the predominantly encountered enteric species (*Escherichia coli*, *Klebsiella oxytoca, Klebsiella pneumoniae, Proteus mirabilis, Morganella morganii* and *Serratia marcescens*), and *Pseudomonas aeruginosa* and *Acinetobacter* spp.

The antibiotics studied are listed in Tables 2,3,4,5. Among *E. coli*, putative ESBL production was defined as those isolates that were intermediate or resistant (non-susceptible) to ceftazidime [17]. Given the large number of isolate results included in the majority of analyses in this study, statistical analysis was not performed, as even subtle differences in percent resistance (<1%) to an antimicrobial agent for any time period or demographic parameters would be reported as highly significant (*P* <0.001).

**Table 2:** *S. aureus*, Coagulase-negative staphylococci, *E. faecalis*, and *E. faecium* isolated from ICU patients during 2000–2002

		United States			Canada			Italy			Germany			France^a^		
						
Organism	Agent	Total n	%S	%R	Total n	%S	%R	Total n	%S	%R	Total n	%S	%R	Total n	%S	%R
*Staphylococcus aureus*	Ampicillin	19,703	6.7	93.3	3,792	12.6	87.4	1,665	5.6	94.4	2,867	16.2	83.8	15	6.7	93.3
	Cefepime	1,260	52.9	46.9	NT^b^	NT	NT	304	15.8	84.2	483	80.5	17.0	<10	NA^c>^	NA
	Cefotaxime	6,898	50.2	49.7	220	55.5	44.5	671	36.4	63.6	729	92.0	8.0	490	63.9	36.1
	Ceftriaxone	5,914	45.6	54.3	153	69.3	30.7	1,048	28.1	71.8	220	88.6	11.4	23	73.9	26.1
	Ciprofloxacin	24,350	47.4	51.0	5,958	74.5	24.1	4,600	39.7	58.6	5,243	73.4	26.1	316	57.0	40.5
	Gentamicin	35,034	85.6	13.7	6,641	89.4	10.3	5,531	40.9	58.0	5,735	90.0	9.7	10,100	90.4	9.4
	Oxacillin	44,939	47.7	52.3	10,105	80.3	19.7	6,147	40.6	59.4	6,475	79.0	21.0	10,512	59.4	40.6
	Teicoplanin	NT	NT	NT	NT	NT	NT	5,868	100	0	4,632	99.8	0.2	8,232	100	0
	Vancomycin	43,245	100	0	7,882	100	0	5,937	100	0	5,276	100	0	9,453	100	0
*Staphylococcus aureus*																
OSSA	Ampicillin	9,047	14.5	85.5	3,055	15.7	84.3	741	12.6	87.4	2,414	19.3	80.7	10	0	100
	Cefepime	672	99.1	0.4	NT	NT	NT	49	98.0	2.0	387	99.5	0.3	NT	NT	NT
	Cefotaxime	3,451	99.7	0.2	122	100	0	244	100	0	653	100	0	312	100	0
	Ceftriaxone	2,707	99.5	0.2	106	100	0	295	99.0	0.3	194	100	0	16	100	0
	Ciprofloxacin	11,827	91.2	6.5	4,692	93.5	4.8	1,902	91.4	4.9	4,171	91.4	8.0	188	90.4	6.4
	Gentamicin	16,951	98.3	1.4	5,384	98.1	1.8	2,223	95.1	4.5	4,527	98.4	1.5	5,958	99.4	0.5
	Oxacillin	21,416	100	0	8,110	100	0	2,495	100	0	5,115	100	0	6,244	100	0
	Teicoplanin	NT	NT	NT	NT	NT	NT	2,402	100	0	3,593	99.9	0.1	5,018	100	0
	Vancomycin	20,110	100	0	6,046	100	0	2,430	100	0	4,002	100	0	5,580	100	0
*Staphylococcus aureus*																
ORSA	Ampicillin	10,656	0	100	737	0	100	924	0	100	453	0	100	<10	NA	NA
	Cefepime	588	0	100	NT	NT	NT	255	0	100	96	4.2	84.4	<10	NA	NA
	Cefotaxime	3,447	0.6	99.3	98	0	100	427	0	100	76	23.7	76.3	178	0.6	99.4
	Ceftriaxone	3,207	0	100	47	0	100	753	0.3	99.7	26	3.8	96.2	<10	NA	NA
	Ciprofloxacin	12,523	6.1	93.1	1,266	3.9	95.5	2,698	3.3	96.4	1,072	3.3	96.6	128	7.8	90.6
	Gentamicin	18,083	73.7	25.2	1,257	52.0	46.8	3,308	4.5	94.0	1,208	58.7	40.5	4,142	77.5	22.2
	Oxacillin	23,523	0	100	1,995	0.2	99.8	3,652	0	100	1,360	0	100	4,268	0	100
	Teicoplanin	NT	NT	NT	NT	NT	NT	3,466	100	0	1,039	99.7	0.3	3,214	100	0
	Vancomycin	23,135	100	0	1,836	100	0	3,507	100	0	1,274	100	0	3,873	100	0
*Staphylcoccus* species, coagulase-negative																
	Ampicillin	16,288	5.7	94.3	3,533	6.3	93.7	2,142	10.6	89.4	4,075	8.1	91.9	<10	NA	NA
	Cefepime	991	11.8	88.1	<10	NA	NA	116	0	100	625	11.0	73.1	<10	NA	NA
	Cefotaxime	5,538	17.7	82.3	240	17.9	82.1	335	16.7	83.3	625	37.4	62.4	174	28.7	69.0
	Ceftriaxone	3,471	14.8	84.8	116	22.4	77.6	512	11.7	88.3	412	25.0	74.8	<10	NA	NA
	Ciprofloxacin	18,829	40.2	58.3	5,366	44.4	54.7	5,102	42.7	54.0	6,197	29.5	67.6	198	44.4	53.0
	Gentamicin	27,248	51.5	38.1	5,571	40.6	47.3	5,241	33.8	60.7	6,848	41.5	51.7	9,422	46.8	51.5
	Oxacillin	35,135	15.8	84.2	9,172	20.6	79.4	5,961	15.2	84.8	7,442	18.6	81.4	9,884	30.1	69.9
	Teicoplanin	NT	NT	NT	NT	NT	NT	5,797	93.7	2.4	5,096	95.6	0.7	7,670	84.6	3.1
	Vancomycin	34,424	100	0	8,239	100	0	5,937	100	0	6,953	100	0	8,300	100	0
*Staphylcoccus* species, coagulase-negative																
Oxacillin susceptible	Ampicillin	2,582	35.7	64.3	638	34.6	65.4	437	51.7	48.3	824	39.6	60.4	<10	NA	NA
	Cefepime	117	100	0	NT	NT	NT	NT	NT	NT	<10	NA	NA	NT	NT	NT
	Cefotaxime	978	99.5	0.2	42	100	0	56	100	0	128	100	0	54	92.6	0
	Ceftriaxone	523	98.3	0.4	26	100	0	59	100	0	103	100	0	<10	NA	NA
	Ciprofloxacin	2,844	82.4	16.6	988	91.8	7.6	779	87.7	10.1	1,103	89.5	9.2	78	83.3	14.1
	Gentamicin	4,424	93.5	4.2	1,068	91.9	5.3	698	94.3	5.3	1,263	96.5	2.7	2,822	93.9	5.4
	Oxacillin	5,565	100	0	1,886	99.9	0.1	904	100	0	1,383	100	0	2,980	100	0
	Teicoplanin	NT	NT	NT	NT	NT	NT	890	99.1	0.3	691	98.4	0.3	2,454	95.8	0.2
	Vancomycin	5,240	100	0	1,587	100	0	897	100	0	981	100	0	2,467	100	0
*Staphylcoccus* species, coagulase-negative																
Oxacillin resistant	Ampicillin	13,706	0.1	99.9	2,895	0	100	1,705	0	100	3,251	0.2	99.8	<10	NA	NA
	Cefepime	874	0	99.9	<10	NA	NA	116	0	100	624	10.9	73.2	<10	NA	NA
	Cefotaxime	4,560	0.1	99.9	198	0.5	99.5	279	0	100	497	21.3	78.5	120	0	100
	Ceftriaxone	2,948	0	99.8	90	0	100	453	0.2	99.8	309	0.0	99.7	<10	NA	NA
	Ciprofloxacin	15,985	32.7	65.8	4,378	33.7	65.3	4,323	34.7	61.9	5,094	16.5	80.2	120	19.2	78.3
	Gentamicin	22,824	43.3	44.7	4,503	28.5	57.3	4,543	24.5	69.2	5,585	29.1	62.8	6,600	26.6	71.3
	Oxacillin	29,570	0	100	7,286	0	100	5,057	0	100	6,059	0	100	6,904	0	100
	Teicoplanin	NT	NT	NT	NT	NT	NT	4,907	92.7	2.8	4,405	95.1	0.8	5,216	79.3	4.5
	Vancomycin	29,184	100	0	6,652	100	0	5,040	100	0	5,972	100	0	5,833	100	0
*Enterococcus faecalis*																
	Ampicillin	7,865	98.8	1.2	1,000	99.4	0.6	1,289	95.3	4.7	1,902	99.6	0.4	1,183	99.5	0.2
	Ciprofloxacin	3,311	56.9	38.7	625	45.3	50.4	1,159	64.0	31.1	2,012	39.7	39.5	559	78.5	17.0
	Gentamicin (HL Testing)	5,503	65.1	34.8	706	63.0	36.8	1,156	62.9	37.1	965	64.8	35.2	1,563	63.6	13.4
	Teicoplanin	NT	NT	NT	<10	NA	NA	1,248	97.1	2.4	1,294	99.7	0.2	1,747	99.9	0.1
	Vancomycin	7,656	95.1	4.5	1,005	98.3	0.9	1,303	96.7	2.8	1,636	99.4	0.3	1,811	99.7	0.2
*Enterococcus faecium*																
	Ampicillin	3,896	9.7	90.3	383	17.2	82.8	260	21.5	78.5	481	12.3	87.7	151	41.7	49.7
	Ciprofloxacin	1,846	5.3	92.5	221	10.9	85.5	234	10.3	77.4	591	6.9	73.9	66	21.2	39.4
	Gentamicin (HL Testing)	2,512	57.5	42.5	291	59.5	40.5	223	67.7	32.3	349	60.2	39.8	263	65.4	12.2
	Teicoplanin	23	8.7	87.0	<10	NA	NA	234	86.3	13.7	517	97.9	2.1	266	99.6	0.4
Vancomycin	4,066	23.2	76.3	415	85.1	14.5	264	75.4	24.2	628	93.9	4.8	247	98.4	0.8	

^a^NCCLS breakpoints were used for all countries, except (CA-SFM) ^b^Not tested ^c^Not applicable if <10 isolates were tested

**Table 3:** *S. pneumoniae*, *S. pyogenes*, *S. agalactiae*, and Viridans group streptococci isolated from ICU patients during 2000–2002

		United States			Canada			Italy			Germany			France^a^		
						
Organism	Agent	Total n	%S	%R	Total n	%S	%R	Total n	%S	%R	Total n	%S	%R	Total n	%S	%R
*Streptococcus pneumoniae*	Amoxicillin	120	91.7	2.5	31	100	0	60	93.3	6.7	17	100	0	1,328	71.2	2.3
	Cefepime	22	90.9	4.5	25	60.0	12.0	66	90.9	7.6	NT^b^	NT	NT	<10	NA^c^	NA
	Cefotaxime	1,571	82.2	6.3	145	93.8	0.7	108	93.5	4.6	63	100	0	1,181	77.1	0.8
	Ceftriaxone	2,373	88.3	3.2	145	91.7	0.7	145	91.7	3.4	29	100	0	544	80.1	0.6
	Clarithromycin	184	71.7	25.5	56	69.6	30.4	90	64.4	31.1	<10	NA	NA	NT	NT	NT
	Erythromycin	3,029	67.9	30.5	539	78.5	20.8	313	69.6	28.1	405	88.6	9.4	1,567	59.0	38.8
	Levofloxacin	2,133	99.1	0.4	356	98.6	1.1	174	98.3	0.6	340	99.4	0.3	62	98.4	1.6
	Penicillin	3,096	51.5	20.2	325	59.1	7.1	198	77.3	7.6	102	96.1	2.0	1,387	45.5	17.9
	Vancomycin	2,865	100	-^c^	271	100	-	231	100	-	190	100	-	1,479	100	-
*Streptococcus pyogenes*																
	Amoxicillin	NT	NT	NT	NT	NT	NT	NT	NT	NT	NT	NT	NT	58	100	0
	Cefepime	<10	NA	NA	NT	NT	NT	NT	NT	NT	NT	NT	NT	NT	NT	NT
	Cefotaxime	32	100	-	29	100	-	<10	NA	NA	11	100	-	30	100	-
	Ceftriaxone	75	100	-	<10	NA	NA	<10	NA	NA	<10	NA	NA	<10	NA	NA
	Clarithromycin	19	84.2	5.3	<10	NA	NA	17	88.2	11.8	NT	NT	NT	NT	NT	NT
	Erythromycin	118	92.4	6.8	102	81.4	11.8	59	74.6	23.7	63	84.1	11.1	170	82.9	14.7
	Levofloxacin	71	97.2	1.4	<10	NA	NA	<10	NA	NA	61	77.0	4.9	NT	NT	NT
	Penicillin	140	100	-	97	100	-	58	100	-	64	100	-	139	100	-
	Vancomycin	121	100	-	42	100	-	12	100	-	34	100	-	162	100	-
*Streptococcus agalactiae*																
	Amoxicillin	NT	NT	NT	NT	NT	NT	NT	NT	NT	NT	NT	NT	165	100	0
	Cefepime	28	100	-	NT	NT	NT	<10	NA	NA	NT	NT	NT	NT	NT	NT
	Cefotaxime	71	100	-	17	100	-	24	100	-	50	100	-	50	100	-
	Ceftriaxone	184	100	-	<10	NA	NA	38	100	-	37	100	-	<10	NA	NA
	Clarithromycin	21	81.0	9.5	<10	NA	NA	21	71.4	28.6	NT	NT	NT	<10	NA	NA
	Erythromycin	489	76.3	21.7	222	82.9	14.9	121	77.7	18.2	192	83.9	10.9	588	79.9	16.2
	Levofloxacin	333	97.9	1.2	<10	NA	NA	51	98.0	0	180	91.1	1.7	173	99.4	0
	Penicillin	518	100	-	226	100	-	145	100	-	184	100	-	369	100	-
	Vancomycin	463	100	-	179	100	-	143	100	-	65	100	-	526	100	-
*Streptococcus* viridans group																
	Amoxicillin	NT	NT	NT	NT	NT	NT	NT	NT	NT	NT	NT	NT	268	92.9	0.7
	Cefepime	23	95.7	4.3	NT	NT	NT	12	66.7	33.3	NT	NT	NT	NT	NT	NT
	Cefotaxime	434	83.6	11.1	101	92.1	4.0	31	90.3	9.7	75	97.3	2.7	56	94.6	0
	Ceftriaxone	678	87.3	7.7	130	89.2	3.8	99	81.8	18.2	40	97.5	2.5	<10	NA	NA
	Clarithromycin	34	52.9	38.2	21	76.2	19.0	21	71.4	23.8	<10	NA	NA	NT	NT	NT
	Erythromycin	959	57.2	37.7	289	71.6	23.2	192	64.6	32.8	796	88.1	9.2	626	59.9	31.6
	Levofloxacin	331	96.1	2.7	<10	NA	NA	16	87.5	0	93	89.2	4.3	<10	NA	NA
	Penicillin	1,047	63.7	6.2	303	79.2	0	61	78.7	8.2	<10	NA	NA	452	69.0	3.1
Vancomycin	1,095	100	-	276	100	-	180	100	-	277	100	-	580	100	-	

^a^NCCLS breakpoints were used for all countries, except France (CA-SFM) ^b^Not tested ^c^Breakpoints do not currently exist to interpret as S (susceptible) or R (resistant)

**Table 4:** *Enterobacteriaceae* isolated from ICU patients during 2000–2002

		United States			Canada			Italy			Germany			France^a^		
						
Organism	Agent	Total n	%S	%R	Total n	%S	%R	Total n	%S	%R	Total n	%S	%R	Total n	%S	%R
*Escherichia coli*	Cefepime	10,356	98.1	1.5	207	98.1	1.9	1,426	98.1	1.4	2,830	98.6	1.2	4,358	98.9	0.6
	Cefotaxime	9,086	96.5	2.2	3,231	96.3	2.5	1,748	94.5	3.8	5,828	97.8	1.8	9,362	98.8	0.6
	Ceftazidime	14,574	95.3	3.0	4,438	97.7	1.6	2,548	94.7	3.7	3,924	97.9	1.6	9,164	97.8	1.2
	Ceftriaxone	15,897	97.4	1.7	3,829	96.8	2.2	1,423	94.4	4.2	534	99.8	0.2	834	98.6	1.0
	Ciprofloxacin	17,294	89.0	10.7	5,028	90.3	9.5	2,616	87.0	12.7	4,615	86.7	12.4	8,577	93.1	6.5
	Gentamicin	20,581	92.4	6.5	6,654	92.8	5.3	2,650	92.2	6.6	4,825	94.3	5.2	9,442	95.4	4.2
	Imipenem	15,353	100	0	3,386	100	0	2,254	100	0	5,172	100	0	8,994	100	0
	Levofloxacin	14,920	88.2	11.6	776	85.1	13.9	496	86.5	13.3	3,137	88.2	11.0	NT^b^	NT	NT
	Piperacillin-tazobactam	13,573	93.1	3.6	4,305	95.1	2.4	1,879	95.8	2.4	5,637	93.6	3.4	7,255	95.4	1.1
	Trimethoprim-sulfamethoxazole	20,296	79.2	20.7	6,737	84.6	15.3	2,440	75.0	24.9	5,598	73.1	26.6	9,028	78.2	21.1
*Klebsiella oxytoca*																
	Cefepime	1,476	96.2	3.3	19	100	0	255	99.6	0	566	96.8	2.7	478	97.1	0.4
	Cefotaxime	1,324	92.7	4.7	486	94.2	4.5	230	96.5	1.7	1,117	93.8	4.4	865	96.3	0.8
	Ceftazidime	1,909	91.7	7.0	661	94.9	4.1	361	83.4	15.2	749	95.3	4.5	870	98.3	0.5
	Ceftriaxone	2,035	89.9	6.6	536	93.8	2.8	197	81.7	2.0	83	97.6	0	79	87.3	2.5
	Ciprofloxacin	2,226	92.5	5.9	745	96.0	3.0	368	96.7	3.0	905	90.1	7.8	815	94.5	4.8
	Gentamicin	2,569	89.9	8.3	857	95.0	4.9	366	89.6	3.0	1,016	98.2	1.2	865	97.1	2.4
	Imipenem	2,061	100	0	516	100	0	337	100	0	1,062	100	0	845	100	0
	Levofloxacin	1,754	93.3	3.4	159	96.9	1.3	133	97.0	3.0	560	94.6	3.2	NT	NT	NT
	Piperacillin-tazobactam	1,801	82.7	13.9	624	91.2	7.1	313	81.8	11.2	1,113	78.9	18.1	742	88.3	10.4
	Trimethoprim-sulfamethoxazole	2,467	92.5	7.5	863	96.3	3.6	308	95.1	4.9	1,084	93.7	6.3	802	94.1	5.7
*Klebsiella pneumoniae*																
	Cefepime	7,276	95.8	3.4	98	100	0	552	93.5	5.6	1,068	95.7	3.5	840	95.6	3.0
	Cefotaxime	6,243	91.0	6.1	1,411	97.9	1.5	850	76.7	16.4	2,414	93.1	6.0	1,553	94.4	1.9
	Ceftazidime	9,597	88.5	10.1	2,238	97.5	2.2	1,142	69.8	28.5	1,665	90.0	8.2	1,591	92.5	5.2
	Ceftriaxone	10,337	92.7	4.7	1,736	97.9	1.1	816	75.2	15.0	166	98.8	0.6	112	86.6	5.4
	Ciprofloxacin	11,089	89.9	8.4	2,484	91.8	7.2	1,190	88.2	9.9	2,128	85.4	9.4	1,473	89.5	8.7
	Gentamicin	13,012	91.6	7.0	2,906	96.7	2.9	1,211	81.4	14.5	2,065	91.6	6.1	1,553	97.1	2.7
	Imipenem	10,263	100	0	1,766	100	0	1,066	100	0	2,351	100	0	1,567	100	0
	Levofloxacin	9,626	91.0	6.4	485	93.4	3.7	287	78.4	21.3	1,228	92.6	4.4	NT	NT	NT
	Piperacillin-tazobactam	9,359	85.9	7.4	2,160	91.5	2.7	746	82.2	14.6	2,408	84.9	8.3	1,286	89.4	5.1
	Trimethoprim-sulfamethoxazole	12,641	88.6	11.1	2,924	92.8	7.1	1,103	82.0	18.0	2,324	82.2	17.2	1,443	88.2	10.9
*Morganella morganii*																
	Cefepime	566	95.9	2.3	<10	NA	NA	121	97.5	2.5	262	94.7	5.0	412	96.1	0.2
	Cefotaxime	499	78.8	8.4	156	91.0	3.8	144	74.3	6.3	437	86.7	3.9	678	81.1	5.9
	Ceftazidime	715	73.6	17.3	256	79.7	10.9	213	75.6	15.0	313	84.0	7.7	673	78.6	8.0
	Ceftriaxone	806	91.1	2.2	219	96.3	1.4	125	91.2	3.2	22	86.4	0	57	84.2	5.3
	Ciprofloxacin	841	78.1	20.7	292	94.2	4.5	220	87.3	9.5	344	97.7	2.0	634	88.6	8.5
	Gentamicin	967	84.0	14.1	329	94.5	4.6	222	90.1	8.6	378	96.8	2.1	679	95.6	3.4
	Imipenem	784	100	0	196	100	0	206	100	0	402	100	0	649	99.8	0
	Levofloxacin	725	78.1	19.3	42	95.2	4.8	55	90.9	9.1	251	98.0	1.6	NT	NT	NT
	Piperacillin-tazobactam	725	91.2	5.1	254	97.2	1.6	150	94.0	3.3	430	94.2	3.5	564	91.0	4.6
	Trimethoprim-sulfamethoxazole	936	75.1	24.7	329	91.8	8.2	193	79.8	20.2	435	93.1	6.9	627	83.9	14.2
*Proteus mirabilis*																
	Cefepime	1,964	98.2	1.0	20	100	0	395	87.6	11.4	599	99.2	0.8	736	99.0	0.1
	Cefotaxime	1,794	99.1	0.5	295	99.7	0	441	69.4	23.4	1,209	98.8	0.7	1,503	99.5	0.1
	Ceftazidime	2,684	98.0	1.1	463	99.4	0.2	630	86.0	9.4	821	98.5	1.0	1,505	99.3	0.2
	Ceftriaxone	3,034	99.4	0.3	392	99.5	0	385	80.5	13.8	77	98.7	0	72	100	0
	Ciprofloxacin	3,169	85.2	12.7	504	95.2	4.6	657	70.6	22.7	980	92.9	5.1	1,424	90.9	6.8
	Gentamicin	3,796	91.5	7.7	698	92.6	7.2	670	61.6	37.2	992	92.9	5.9	1,509	91.3	7.9
	Imipenem	2,850	100	0	367	100	0	580	100	0	1,020	100	0	1,319	100	0
	Levofloxacin	2,825	87.8	10.5	94	100	0	202	61.9	34.7	688	96.5	2.3	<10	NA^c^	NA
	Piperacillin-tazobactam	2,715	97.7	0.8	449	98.2	0.2	465	95.7	2.8	1,201	98.6	0.8	1,231	99.3	0.2
	Trimethoprim-sulfamethoxazole	3,706	85.2	14.7	708	89.4	10.6	615	61.6	38.0	1,159	80.8	19.1	1,411	79.7	18.6
*Serratia marcescens*																
	Cefepime	3,653	96.7	2.3	52	96.2	1.9	497	96.8	2.2	546	94.1	3.5	509	98.6	0.2
	Cefotaxime	3,134	87.0	5.7	670	92.8	2.7	470	79.6	9.8	951	84.0	7.5	809	81.5	3.3
	Ceftazidime	4,718	89.7	7.9	1,113	95.2	3.0	738	81.4	13.3	851	89.7	7.5	812	94.7	3.0
	Ceftriaxone	4,710	90.5	4.6	846	95.4	1.7	444	86.7	6.3	160	45.6	0	115	77.4	4.3
	Ciprofloxacin	5,006	91.0	6.7	1,292	85.0	11.7	757	83.5	4.5	978	72.6	12.4	787	78.9	10.5
	Gentamicin	5,905	92.9	5.9	1,313	94.6	5.2	758	97.4	2.1	665	92.9	6.3	808	91.6	6.6
	Imipenem	4,960	100	0	880	100	0	727	100	0	1,018	100	0	805	100	0
	Levofloxacin	4,356	94.3	4.2	264	92.4	4.2	266	95.5	1.5	595	87.6	6.6	<10	NA	NA
	Piperacillin-tazobactam	4,337	88.1	5.1	1,155	91.6	3.3	547	92.7	3.8	1,053	77.6	3.1	749	82.6	2.4
Trimethoprim-sulfamethoxazole	5,697	95.9	3.9	1,325	94.9	5.1	646	81.4	18.6	908	88.1	10.9	699	84.1	13.6	

^a^NCCLS breakpoints were used for all countries, except France (CA-SFM) ^b^Not tested ^c^Not applicable if <10 isolates were tested

**Table 5:** *P. aeruginosa* and *Acinetobacter* spp isolated from ICU patients during 2000–2002

		United States			Canada			Italy			Germany			France		
						
Organism	Agent	Total n	%S	%R	Total n	%S	%R	Total n	%S	%R	Total n	%S	%R	Total n	%S	%R
*Acinetobacter species*	Cefepime	5,162	43.8	40.2	97	67.0	23.7	475	17.9	73.7	623	74.2	10.8	857	28.0	40.3
	Cefotaxime	3,830	23.3	49.9	705	36.7	34.9	555	11.0	78.7	1,254	34.9	24.6	671	15.4	38.7
	Ceftazidime	5,954	42.2	40.8	1,162	70.8	22.9	692	25.6	68.5	988	66.7	14.5	1,106	34.9	35.5
	Ceftriaxone	4,709	16.3	55.9	874	32.4	28.7	452	8.8	72.6	104	42.3	11.5	81	9.9	51.9
	Ciprofloxacin	5,808	39.7	58.0	1,156	72.1	25.9	686	21.1	76.7	1,126	74.8	22.9	1,038	37.7	61.2
	Gentamicin	6,618	47.2	47.2	1,185	72.8	22.8	768	23.3	72.4	979	82.0	14.1	936	49.3	43.5
	Imipenem	6,006	87.0	7.5	918	95.8	1.9	569	77.9	19.0	1,253	96.2	3.4	1,088	93.8	3.8
	Levofloxacin	5,099	43.8	52.2	489	61.1	25.6	295	13.9	75.3	840	82.0	10.5	NT^b^	NT	NT
	Meropenem	2,154	66.3	26.5	348	93.7	4.9	455	74.5	13.6	1,024	96.0	3.4	147	68.0	28.6
	Piperacillin	4,658	35.4	45.9	959	66.5	19.5	635	19.5	69.9	1,171	59.7	12.9	805	35.0	50.3
	Piperacillin-tazobactam	3,429	53.6	28.5	903	70.7	23.1	425	35.1	46.4	1,225	81.8	7.5	878	74.5	10.5
	Trimethoprim-sulfamethoxazole	5,697	51.4	48.4	1,155	74.8	25.2	750	44.1	55.7	1,234	83.6	15.6	93	45.2	52.7
*Pseudomonas aeruginosa*																
	Cefepime	20,220	72.5	12.4	371	73.3	12.4	5,056	58.9	28.9	3,483	80.3	7.8	7,967	52.6	16.2
	Cefotaxime	11,283	9.2	50.4	1,836	13.3	47.5	4,181	6.0	70.7	2,689	7.7	52.2	NT	NT	NT
	Ceftazidime	26,353	71.2	17.4	6,036	73.7	13.4	7,640	56.7	31.3	5,141	76.2	14.9	8,547	70.2	14.9
	Ceftriaxone	14,066	12.1	56.4	2,847	11.3	59.7	3,383	8.4	70.4	154	26.6	7.8	NT	NT	NT
	Ciprofloxacin	26,700	62.8	33.1	5,924	67.2	30.2	7,388	58.4	38.8	4,746	68.6	24.4	8,560	55.3	40.6
	Gentamicin	29,268	69.4	21.5	5,951	72.2	15.9	7,522	52.2	41.7	3,913	74.0	14.3	7,327	44.0	46.1
	Imipenem	26,076	73.5	22.1	3,775	77.9	18.2	7,057	59.7	27.8	4,412	70.5	19.0	8,575	69.5	21.4
	Levofloxacin	21,059	62.7	31.7	713	56.8	33.5	2,427	44.9	51.0	2,953	68.0	23.9	NT	NT	NT
	Meropenem	7,540	76.0	18.2	1,266	80.3	14.5	4,082	57.3	32.7	4,351	77.8	13.8	1,818	81.1	6.4
	Piperacillin	22,855	77.7	22.2	5,520	80.9	18.8	7,004	63.1	36.7	4,554	81.7	14.1	8,454	64.1	24.1
	Piperacillin-tazobactam	21,848	85.5	14.4	4,190	91.0	9.0	5,252	77.7	22.0	4,746	85.8	10.7	8,256	69.6	15.9
Trimethoprim-sulfamethoxazole	15,618	3.6	96.4	4,283	4.0	96.0	7,054	4.1	95.8	3,375	4.2	95.8	NT	NT	NT	

^a^NCCLS breakpoints were used for all countries, except France (CA-SFM) ^b^NT = not tested

## Results

In vitro susceptibility data from over 220,000 isolates from ICUs in five countries over the period 2000–2002 were assimilated. The most frequent species isolated from infections in the ICU was *S. aureus*, being most common in three of the five countries (Table 1). The oxacillin resistance rates among *S. aureus* varied markedly across countries from 19.7% in Canada to 59.5% in Italy. *E. coli* (7.7%–15.5%) and *P. aeruginosa* (10.8%–22.3%) were the most frequent Gram-negative organisms encountered. The Gram-positive genus *Enterococcus*, either as *E. faecalis, E. faecium* or non-speciated isolates accounted for <10% of isolates in most countries with *E. faecalis* being the most common species <4.3%. Community-acquired respiratory pathogens such as *Streptococcus pneumoniae* and *Haemophilus influenzae* were relatively uncommon in all five countries.

**Table 1:** Incidence of pathogens isolated from ICU patients by country (%)

United States		Canada		Italy		Germany		France	
				
Organism	Incidence (%)	Organism	Incidence (%)	Organism	Incidence (%)	Organism	Incidence (%)	Organism	Incidence (%)
*S. aureus*^a^	20.2	*S. aureus*^a^	17.4	*P. aeruginosa*	22.3	CNS	16.4	*S. aureus*^1^	17.2
CNS^b^	15.9	CNS	16.1	CNS	18.7	*S. aureus*^a^	13.6	CNS	16.7
*P. aeruginosa*	13.1	*E. coli*	12.6	*S. aureus*^a^	18.1	*E. coli*	12.3	*E. coli*	15.5
*E. coli*	9.2	*P. aeruginosa*	11.3	*E. coli*	7.7	*P. aeruginosa*	10.8	*P. aeruginosa*	13.8
*K. pneumoniae*	5.8	*Enterococcus* spp	7.6	*E. faecalis*	3.9	*Enterococcus* spp	7.4	S. pneumoniae	3.3
*Enterococcus* spp	5.4	*K. pneumoniae*	5.5	*K. pneumoniae*	3.5	*K. pneumoniae*	5.4	*E. cloacae*	3.3
*E. cloacae*	4.3	*E. cloacae*	4.2	*Enterococcus* spp	3.3	*E. cloacae*	4.7	*E. faecalis*	3.0
*E. faecalis*	3.7	*S. marcenscens*	2.5	*E. cloacae*	2.6	E. faecalis	4.3	*K. pneumoniae*	2.7
*S. marcescens*	2.7	*H. influenzae*	2.1	*S. marcescens*	2.2	*P. mirabilis*	2.6	*P. mirabilis*	2.5
*A. baumanii*	2.6	*E. faecalis*	2.1	*P. mirabilis*	1.9	*K. oxytoca*	2.4	*Enterococcus* spp	2.3
*Enterobacteriaceae*^c^ (all species combined)	29.5	*Enterobacteriaceae* (all species combined)	33.0	*Enterobacteriaceae* (all species combined)	30.2	*Enterobacteriaceae* (all species combined)	36.0	*Enterobacteriaceae* (all species combined)	32.1
Total (n)	26,624	Total (n)	54,445	Total (n)	34,609	Total (n)	48,385	Total (n)	62,459

^a^Proportion of *S. aureus* testing as MRSA was USA (52.3%), Canada (19.7%), Italy (59.4%), Germany (21.0%), and France (40.6%) ^b^CNS = Coagulase-negative staphylococci ^c^*Enterobacteriaceae* includes all species of genera occurring at >0.1%

Tables 2,3,4,5 show the antimicrobial susceptibility profiles of various Gram-positive and Gram-negative pathogens isolated from ICU patients against a range of relevant antimicrobials.

Specifically notable susceptibility patterns include the vancomycin susceptibility of all strains of staphylococci. Generally, there was a low proportion of vancomycin resistant *E. faecalis* <5%, whereas vancomycin-resistant *E. faecium* was more prevalent ranging from 0.8% in France to 76.3% in the United States, with a wide inter-country variation (Table 2). Penicillin resistance rates varied among *S. pneumoniae*, from 2.0% in Germany to 20.2% in the US with concurrent ceftriaxone resistance rates of 0% in Germany to 3.4% in Italy (Table 3).

β-lactam activity was assessed by comparing four different cephalosporins and a β-lactam/β-lactamase inhibitor combination, piperacillin-tazobactam. Overall, the putative production of ESBLs among *E. coli* was low, <6%, but ceftazidime resistance was reported at higher rates in *K. pneumoniae* and *S. marcescens*, with the highest rates seen in *M. morganii*, from 16.0% in Germany to 26.4% in the United States (Table 4). Among the gram-negative organisms tested, ceftriaxone resistance rates were usually lower than ceftazidime, with the exception among *P. aeruginosa* and *Acinetobacter* spp. Cefepime, a fourth generation cephalosporin with anti-pseudomonal activity was also more active than ceftazidime (Table 5). Against the *Enterobacteriaceae*, the β-lactam combination agent piperacillin-tazobactam was generally less active than ceftriaxone. These species showed a wide variation in fluoroquinolone susceptibility among both species and countries. Gentamicin resistance rates among the *Enterobacteriaceae* varied from 1.2% among *K. oxytoca* from Germany to 37.2% in *P. mirabilis* from Italy. Ciprofloxacin resistance rates among *E. coli* ranged from 6.5% in France to 12.7% in Italy. Variable fluoroquinolone resistance rates among *S. marcescens* were also demonstrated, with a range of resistance from 4.5% in Italy to 12.4% in Germany.

## Discussion

Data derived from international surveillance studies, such as those presented here, can provide a unique contemporary perspective on the susceptibility of commonly encountered organisms to commonly used antibiotics. Such surveillance systems play a crucial role in detecting emerging trends in resistance. Comparisons of these with data of other recent surveillance programs show the wide variations in susceptibility profiles and the need for ongoing unit-specific surveys.

In Germany the prevalence of resistance among gram-positive organisms remained comparatively low with an incidence of 21% MRSA. In 2000, Frank et al. reported that 96% of German isolates of *S. marcescens* and *M. morganii* were susceptible to ceftazidime, yet in this study we found 89.7% and 84.0%, respectively [9]. A similar decrease in activity was noted with *E. coli* and ciprofloxacin between the two studies, 91% in 1996–1997 compared with 86.7% in this study. Marked decreases in susceptibility of *P. aeruginosa* in Germany were also evident, with no agent showing >85.8% susceptibility (piperacillin-tazobactam) compared with most agents having 85%–94% susceptibility in 1996–1997. Changes of 15–20% have been reported with ceftazidime, imipenem, ciprofloxacin and meropenem, while piperacillin-tazobactam has shown the smallest decrease in susceptibility with <6% over the 4-year period. Piperacillin plus or minus tazobactam and cefepime were the most active agents, based on susceptibility, against *P. aeruginosa* in Germany. Conversely, ceftriaxone and imipenem were the most active agents, based on susceptibility, against *Klebsiella* spp., which account for almost 8% of ICU isolates.

Staphylococcal species from French ICU isolates showed a high proportion of oxacillin resistance, 40.6% and 69. 9% of *S. aureus* and coagulase-negative staphylococci spp., respectively. *S. pneumoniae* showed penicillin resistance of 17.9%, higher than the other four countries, although the activity of third-generation cephalosporins, ceftriaxone and cefotaxime, showed only 0.6% and 0.8% resistance, respectively. Despite a lower ceftazidime susceptibility breakpoint compared to NCCLS standards (MIC 4 μg/ml instead of 8 μg/ml) putative ESBL expression were slightly lower in France than in Germany in 2000–2002. Ceftazidime non-susceptibility rates among *E. coli*, *K. oxytoca*, and *P. mirabilis* were ≤ 2.2%; however, ceftazidime non-susceptibility rates among *K. pneumoniae*, *M. morganii* and *S. marcescens* were 7.5%, 21.4%, and 5.3%, respectively. Imipenem was active against all *Enterobacteriaceae*. Against *P. aeruginosa* and *Acinetobacter* spp., imipenem resistance rates were 21.4% and 3.8%, respectively. Previously, a lower imipenem resistance of 24% among French isolates of *P. aeruginosa* was reported [7].

Among the Italian isolates of staphylococci, oxacillin resistance occurred in 59.4% of *S. aureus* and 84.8% of coagulase-negative isolates. This MRSA rate was similar to that reported by Frank et al. from bacteremic isolates in Italy; however, they reported an increase in MRSA from 25% to 55% over the period 1997 to 2001 [18]. Vancomycin resistance rates of 2.8% for *E. faecalis* and 24.2% for *E. faecium* are some of the highest rates recorded in Europe, although still modest compared to rates experienced in the United States; however, teicoplanin was more active with 2.4% and 13.7% of strains being resistant, respectively. Pneumococcal resistance to penicillin and erythromycin was 7.6% and 28.1%, respectively. The impact of alterations in penicillin-binding protein that reduce penicillin susceptibility have less effect on the activity of third-generation cephalosporins such as ceftriaxone with 3.4% and cefotaxime with 4.6% resistance, respectively. *S. pyogenes* was fully susceptible to penicillin; however, 11.8% of isolates were resistant to clarithromycin and 23.7% were resistant to erythromycin.

The proportion of ESBLs was slightly higher in Italy with *E. coli* showing ceftazidime non-susceptibility of 5.3%, whereas *K. pneumoniae* and *K. oxytoca* demonstrated 30.2% and 16.6% ceftazidime non-susceptibility, respectively. Fluoroquinolone resistance rates among the *Enterobacteriaceae*, using ciprofloxacin as a marker, varied from 3.0% for *K. oxytoca* to 22.7% for *P. mirabilis*, and 12.7% for *E. coli*. Thus, among *Enterobacteriaceae*, ciprofloxacin was generally less active than the third-generation cephalosporin, ceftriaxone. *P. aeruginosa* and *Acinetobacter* spp. strains from Italian ICUs demonstrated significant resistance rates. Isolates of *P. aeruginosa* showed resistance rates of >28% for all agents tested except piperacillin-tazobactam. Thus empiric therapy for possible pseudomonal infections will require combination therapy. *Acinetobacter* spp. showed a similar lack of susceptibility except to imipenem and meropenem (19.0% and 13.6% resistant). An increase in fluoroquinolone resistance in *E. coli* and *K. pneumoniae* in bacteremic isolates from Italy was observed during 1997–2001, with rates of 26.7% and 24%, respectively [9]. An increase in ureidopenicillin resistance was noted in *P. aeruginosa* isolates in Italy from 30% to 37% in a 4-year period [9]. This study showed 22.0% piperacillin-tazobactam and 36.7% piperacillin resistance among ICU *P. aeruginosa* isolates.

In Canada oxacillin-resistance among *S. aureus* was noted in 19.7% and coagulase-negative staphylococci in 79.4%. Vancomycin resistance was reported among 0.9% and 14.5% of *E. faecalis* and *E. faecium*, respectively. The lowest rate of penicillin resistance in *S. pneumoniae* in this study was noted from Canada at 7.1%; however, clarithromycin resistance was 30.4%. Ceftriaxone showed 0.7% resistance whereas cefepime exhibited 12.0% resistance among pneumococci from the ICU.

Overall the susceptibility rates for Gram-negative isolates from Canadian ICUs were higher than those in the other four countries examined. A low rate of ESBLs was reported, but there was variable activity of piperacillin-tazobactam which showed >9% resistance among *Klebsiella* spp. and *S. marcescens* tested. The rate of fluoroquinolone resistance was similar to those of other countries with *E. coli* showing 13.9% levofloxacin resistance. Among *Enterobacteriaceae*, <10% of most species were resistant to third-generation cephalosporins tested with the exception of ceftazidime and *M. morganii*. Resistance among *P. aeruginosa* and *Acinetobacter* spp. was generally lower than in other countries apart from Germany. Only piperacillin-tazobactam showed reliable activity against *P. aeruginosa* (9% resistant), while resistance to all other agents was >19%. *Acinetobacter* spp. remained susceptible to only the carbapenems, imipenem and meropenem.

Comparison of the data from Canadian isolates with those from the United States shows some significant differences. This demonstrates the limitations of pooling Canadian and United States data since the differences between the two regions, such as the rate of MRSA, may have some impact on empiric therapy. Data from the NNIS system has previously reported an increasing trend towards resistance within ICUs in the United States [19]. Oxacillin resistance among staphylococci from ICUs in the United States was 52.3% and 84.2% for *S. aureus* and coagulase-negative species, respectively.

This value is identical to that of *S. aureus* and very similar to the CNS data reported by the 1999 NNIS system. The NNIS highlighted a 37% increase in MRSA over the period 1994–98 to 1999, but only a 2% increase among CNS strains [4]. Vancomycin resistance in the United States was observed in 4.5% of *E. faecalis*; however, over 76% *E. faecium* were vancomycin non-susceptible.

Although streptococci are uncommon ICU pathogens they can be rapidly invasive and possibly fatal unless adequate therapeutic approaches are adopted. *S. pneumoniae* in the United States has acquired a range of resistance mechanisms with resistance to penicillin and the macrolides, clarithromycin and erythromycin, being common, 20.2% and 25.5%–30.5% respectively. The newer generation cephalosporins, ceftriaxone, cefotaxime and cefepime showed good activity against pneumococci, 3.2%, 6.3% and 4.5% resistant, respectively. Less than 1.0% of isolates were resistant to levofloxacin. These data are similar to other recent reports [20].

For *Enterobacteriaceae* which account for approximately 30% of all isolates from ICU infections, the incidence of putative ESBLs was low in *E. coli*, 4.7% but ceftazidime non-susceptibility was higher in *K. oxytoca* 8.3%,*K. pneumoniae* 11.5%,*S. marcescens* 10.3% and *M. morganii* 26.4%. These data are consistent with other recent reports [21]. Fluoroquinolone resistance was observed in all *Enterobacteriaceae* tested, in the US for example, resistance rates were as follows, using ciprofloxacin as a marker: *E. coli* 10.7%, *K. oxytoca* 5.9%, *K. pneumoniae* 8.4%, *M. morganii* 20.7%, *P. mirabilis* 12.7% and *S. marcescens* 6.7%. These data show increased fluoroquinolone resistance compared with recent reports [21]. Jones et al. previously reported susceptibility data on ICU pathogens isolated over the period 1998–2001 [22].

Specifically, enteric bacteria showed changes over this time. Fluoroquinolone resistance doubled among *E. coli* isolates from 3.3–5.5% to 10.8–11.4% [22]. This study showed a generally higher level of activity among third-generation cephalosporins than other reports [23], with ceftriaxone showing <10% resistance rates against most species tested. Piperacillin-tazobactam showed less consistent activity with some species being >14% resistant, e.g. *Klebsiella* spp.,*P. aeruginosa*, and *Acinetobacter* spp. present significant therapeutic challenges in ICUs in the United States. With the exception of cefepime, all other tested antimicrobials demonstrated >12% resistance to *P. aeruginosa*, many considerably higher. Piperacillin-tazobactam showed the next lowest resistance rate, 14.4%, with all other agents having rates of 17% or higher. Non-susceptibility to ciprofloxacin among *P. aeruginosa* was 37.2%, higher than in the Neuberger report. Sahm et al. reported a 10% increase in fluoroquinolone resistance among *P. aeruginosa* in the United States, whereas resistance emerged more slowly with the other classes of antimicrobials tested [12]. *Acinetobacter* infections continue to present significant therapeutic challenges due to the extensive resistance mechanisms demonstrated by the >25% resistance shown in Table 5. Only imipenem has any reliable activity against *Acinetobacter* spp. with an 87% susceptibility rate.

There are several implications of these data. It is essential that local surveillance programs be maintained in each country's ICU setting. The local data are vital to the formulary committees as they select appropriate agents to treat infections. There are clear differences among the five countries studied in this report. Although the predominant pathogens are similar, ongoing surveillance is essential to detect the emergence of resistant species. It is clear that certain classes of compounds are losing activity against the ICU pathogens tested. For example, the fluoroquinolones have reduced susceptibility among many Gram-negative species as well as staphylococci; however, the newer class members have enhanced activity against pneumococci. Advanced-generation cephalosporins have variable activity, with ceftriaxone showing consistently good activity against the *Enterobacteriaceae* and some staphylococci. Ceftazidime has lost potency due to the emergence of ESBL enzymes and also has diminished activity against *P. aeruginosa*. Piperacillin-tazobactam is generally active against *P. aeruginosa* in ICUs. The aminoglycoside, gentamicin has shown continued activity against most *Enterobacteriaceae* in all five countries, and modest activity against *S. aureus* but not against CNS strains. The gentamicin susceptibility of *P. aeruginosa* ranged from 44.0% in France to 74.0% in Germany, whereas *Acinetobacter* spp *.* showed more variable gentamicin susceptibility varying from 23.3% in Italy to 82.0% in Germany. These local data should be considered when treating infections in the ICU.

Use of agents with anti-pseudomonal activity such as cefepime, piperacillin-tazobactam or the carbapenems should preferably be reserved for patient types or infections where this pathogen is present or risk factors exist, as per the ATS Community acquired-pneumonia guidelines [24]. A combination of a third-generation cephalosporin such as ceftriaxone with vancomycin may be appropriate for bloodstream infections based upon the NNIS etiology data from 1992–1999.

## Conclusions

The current study confirmed the emergence of fluoroquinolone resistance among various Gram-negative species and staphylococci, which may be increasing due to the heightened use of these drugs; however the reported ESBL rates among *Enterobacteriaceae* was lower than noted in other studies and appeared to be stable. The prevalence of MRSA, perhaps the most significant resistant hospital pathogen, varied among the five countries and appeared to be increasing. Parenteral cephalosporins such as ceftriaxone and cefotaxime remained quite active against *Enterobacteriaceae*. Up-to-date susceptibility data should be made available as rapidly as possible to physicians so that appropriate targeted empirical therapy can be instituted, this approach can assist in maintaining the activity of the current antimicrobials. While local surveillance studies remain crucial, national surveillance studies such as this can provide an invaluable data source to provide guidance in formulary decision-making.

## Authors Contributions

MJ conceived the study, provided data interpretation and drafted the manuscript. DD analyzed the study data; JK and DS provided expert microbiological analysis and interpretation of study data; RW provided clinical expertise in interpretation of data and drafting manuscript. All authors read and approved the final manuscript.
